# Application of Value Framework to Phase III Trials of Immune Checkpoint Inhibitors in Esophageal and Gastric Cancer

**DOI:** 10.1093/oncolo/oyac187

**Published:** 2023-01-18

**Authors:** Rajat Thawani, Neha Agrawal, Nicholas F Taflin, Adel Kardosh, Emerson Y Chen

**Affiliations:** Knight Cancer Institute, Division of Hematology and Oncology, Oregon Health & Science University, Portland, OR 97239, USA; Department of Medicine, Oregon Health & Science University, Portland, OR 97239, USA; Department of Medicine, Oregon Health & Science University, Portland, OR 97239, USA; Knight Cancer Institute, Division of Hematology and Oncology, Oregon Health & Science University, Portland, OR 97239, USA; Knight Cancer Institute, Division of Hematology and Oncology, Oregon Health & Science University, Portland, OR 97239, USA

**Keywords:** esophageal cancer, gastric cancer, comparative effectiveness research, outcome assessment, antineoplastic agents, immunotherapy

## Abstract

**Background:**

Recent trials testing immune-checkpoint inhibitors in esophago-gastric malignancies have shown mixed results. We aim to assess key subgroups using the ASCO Net Health Benefit Score (NHBS) and ESMO Magnitude of Clinical Benefit Scale (MCBS).

**Materials and Methods:**

A search for phase III trials of FDA-approved anti-PD-1 or anti-PD-L1 drugs in esophago-gastric cancer trials was identified using www.clinicaltrials.gov. These published studies were scored using the ASCO NHBS and ESMO MCBS. The ASCO NHBS scores were compared by primary site of cancer (esophageal vs gastric) and PD-L1 expression using the Mann-Whitney test and the ESMO-MCBS grading, by Fisher’s Exact test.

**Results:**

Fifteen of 45 clinical trials were included. Of them, 6 were primarily esophageal cancer trials, and 9 were primarily gastric cancer trials. Ten stratified their analysis based on PD-L1 expression. The ASCO NHBS score was higher (mean 40, range 20 to 56.6 vs. mean 12, range −1.1 to 18.4, *P* < .01) for esophageal cancer than gastric cancer. No difference was observed in survival and response endpoints between the 2 groups. Similarly, the ESMO MCBS scored higher for esophageal cancer group than gastric cancer (*P* < .05). Additionally, the scores were higher in those with high PD-L1 expression vs. low PD-L1 (mean 36, range 11.2-66.6 vs. mean 14, range −19.5 to 43.6, *P* < .05).

**Conclusion:**

The ASCO NHB and ESMO scores were consistently higher among esophageal cancer trials than gastric cancer trials and in those with high PD-L1 expression than low expression. Histology and PD-L1 expression should be considered when discussing value of immunotherapy to patients.

## Teaser

Although survival has improved over the past several decades, esophageal and gastric cancers still have poor survival outcomes. This article reviews the risks and benefits of immunotherapy in different subgroups for treatment of esophageal and gastric cancers.

**Implications for Practice**

In this cohort of drug registration trials of immune-checkpoint inhibitors for upper GI cancers, the ASCO NHB scores and ESMO score support higher clinical benefit from immunotherapy checkpoint inhibitors among to patients with esophageal cancer, many of whom with squamous histology, over gastric cancer, and also those with high PD-L1 expression over low expression. Careful consideration of clinical value of immunotherapy benefit should be discussed with patients per histology and PD-L1 expression.

## Introduction

Advanced and metastatic esophageal and gastric cancer portends poor prognosis with limited durable treatment options. In 2020, GLOBOCAN estimated approximately 544 076 deaths globally from esophageal cancer, which is one of every 18 cancer-related deaths, and 768 793 deaths from stomach cancer, which is one of every 13 cancer-related deaths.^1^ Gastric cancer has the fourth highest number of cancer-related deaths after lung, colorectal, and liver cancers.^1^ While survival has improved over the past several decades, esophageal and gastric cancers still have poor survival outcomes of less than 20% at 5 years.^2,3^

Prior to 2017, cytotoxic chemotherapy regimens were the predominant therapy of choice for metastatic esophageal and gastric cancer. Additional targeted agents focusing on ERBB2/HER2 and VEGF pathways offered additional modest survival advantages.^4,5^ A meta-analysis of most conventional cytotoxic agents in phases II and III trials established fluoropyrimidines, platinums (namely cisplatin and oxaliplatin), and taxanes as the preferred classes of drugs used in advanced esophagogastric cancers.^6^ However, given that esophagogastric cancers were known to have higher tumor mutation burden on average compared to colorectal, pancreatic, and other gastrointestinal (GI) cancers,^7^ immunotherapy checkpoint inhibitors were hypothesized to be efficacious. A number of drug registration trials then led to several drug approvals by the FDA since 2017. Despite some successful drug registration trials in using immunotherapy checkpoint inhibitors, specifically anti-programmed death (PD-1) and cytotoxic T-lymphocyte associated protein 4 (CTLA-4) agents, for esophageal and gastric cancer, there were still notable misses where immunotherapy was no better than conventional chemotherapy, including withdrawal of one notable immunotherapy drug on July 6, 2021 despite initial treatment response of 11.6% in the treatment-refractory patient population.^8^ Both positive and negative clinical trials with wide range of clinical benefit exist for immunotherapy checkpoint inhibitors in esophagogastric cancers, and a systematic review to summarize the clinical nuances and overall benefit would be most informative.

The American Society of Clinical Oncology (ASCO) and European Society for Medical Oncology (ESMO) value frameworks have been studied to assess patient-oriented benefit gleaned from drug registration trials based on strength of primary endpoint, adverse events, and quality of life.^9^ These scores use efficacy outcomes and safety data from the drug registration trials to quantify clinical benefit attributable to these new therapies. The scores vary based on the available survival and response outcomes but preferentially use overall survival, if available, over progression-free survival (PFS) and response rate (RR). Based on the balance between efficacy and tolerability as well as between objective data and symptoms reporting, the framework computes a score that can be used for treatment comparisons. This facilitates shared decision making by patients and oncologists to select treatments that are high value.^10^ Since their validation, more analyses have been done using these methods in variety of cancer types and treatment settings including both curative and palliative scenarios.^11-13^ Currently, there is little known about the risk-benefit ratio informed by the ASCO value framework and ESMO value framework for anti-PD-1 and anti-PD-L1 therapies among all published randomized trials for esophageal and gastric cancer.

The aim of our study was to conduct a review using ASCO value framework, supplemented by ESMO values assessment in weighing risks and benefits of use of immunotherapy use in esophageal and gastric cancer patients.

## Methods

A systematic review of randomized phase III trials that tested anti-PD-1 and anti-PD-L1 agents for esophageal and gastric cancer was conducted. Regardless of statistical significance, we aimed to estimate the magnitude of patient-oriented benefit across these immunotherapy registration trials and to compare the estimated clinical benefit between esophageal and gastric cancer primary and PD-L1 expression.

A detailed search of esophageal and gastric cancer trials was completed on https://clinicaltrials.gov/, which is a public online database maintained by the National Library of Medicine and National Institutes of Health for registering clinical trials. Studies were selected based on the search word “esophageal cancer” and “gastric cancer” as well as FDA-approved anti-PD-1 or PD-L1 immunotherapy drugs such as “pembrolizumab”, “nivolumab”, “avelumab”, “atezolizumab”, “durvalumab”, “cemiplimab”, and “dostarlimab.” Filters for phase III trials were then applied. The list of clinical trials was initially obtained on 2/15/2021 and reviewed again January 2, 2021, at which time “tislelizumab” was recommended to be added, as it was under FDA review for approval in esophageal cancer. The study names and registration numbers were then used to identify primary source data from PubMed, ASCO meeting library, and ESMO meeting library. The final literature search with the selected studies was independently reviewed by and agreed between R.T. and E.Y.C.

All reviewed studies had published articles by February 5, 2022. Only randomized-controlled trials with FDA-approved agents were used. Published literature was preferred over meeting library abstracts and presentations. Data were collected on the selected trials by R.T and E.Y.C. For every selected study, data related to the immunotherapy drug, clinical trial design, relevant dates, key endpoints, and FDA approval process were all extracted. Specific package insert indications and Supplementary material were also verified using drugs@FDA website https://www.accessdata.fda.gov/scripts/cder/daf/index.cfm.

The ASCO Value Framework Net Health Benefit Score version 2 (ASCO-NHB v2) was computed for every included trial. The ESMO-Magnitude of Clinical Benefit Scale version 1.1 (ESMO-MCBS v1.1) was also completed for sensitivity analysis. NHB score per ASCO-NHB v2 was the primary endpoint. Scores from (1) each regimen’s clinical benefit based on the key endpoint with priority given for overall survival (OS) followed by progression-free survival (PFS) then response rate (RR), (2) toxicity profile based on frequency and severity of only symptomatic side effects, and (3) potential bonuses for the tail of the curve, symptom palliation, quality-of-life improvement, and treatment-free intervals, were added to yield the total NHB score. The outcome endpoint that led to the approval indication, which was often the endpoint of the entire randomized cohort was used for (1) clinical benefit score. Other key endpoints with key subgroups were also recorded. (2) Toxicity profile was based on the main tables of the published article rather than package inserts and meeting library abstracts. Both proportions and severity grade contributed to the toxicity scores of each regimen based on ASCO-NHB v2. Asymptomatic lab abnormalities were excluded per ASCO-NHB v2. Insufficient data regarding drug prices prevented inclusion of cost-effective analyses for ASCO-NHB v2. The secondary endpoint was the ESMO-MCBS v1.1, which uses both endpoints and quality of life assessments to input magnitude of clinical benefit (MCB) grades from a 5-point scale (1-5). Procedures and adjustments for adjuvant and metastatic scenarios were followed per ASCO-NHB v2 and ESMO-MCBS v1.1. Details of these worksheets can be found on https://www.asco.org/news-initiatives/current-initiatives/cancer-care-initiatives/value-cancer-care, and these procedures have been previously validated.^10^ For trials with more than one experimental arm, the arm that was in line with first FDA approval indication by February 5, 2022 was used for analysis. Since the FDA approved nivolumab plus ipilimumab for squamous esophageal cancer on May 27, 2022, analysis comparing chemotherapy-free arms was subsequently added for CHECKMATE 648 and KEYNOTE 062.

Both R.T. and E.Y.C. reviewed independently and agreed on the scores computed for every trial. All collected data were from published literature or online websites, possessed no direct protected health information, and so did not meet criteria to be submitted to the local institutional review board. Lists of trials taken from ClinicalTrials.gov, drugs@FDA, and PubMed are all listed in the Supplementary Material.

The ASCO-NHB v2 NHB scores were compared between esophageal and gastric cancer trials using the Mann–Whitney test. The ESMO-MCBS v1.1 grade in the sensitivity analysis were compared using Fisher’s exact test. Study-specific endpoints, such as overall survival (OS), progression-free survival (PFS), and response rate (RR), were also compared between the 2 groups. Key subgroup such as PD-L1 was also analyzed. Other relevant subgroups such as histology (eg, adenocarcinoma vs. squamous) had insufficient data, so only descriptive data was done. PD-L1 expression subgroups (high vs. low) were compared, preferentially using CPS of 10 for pembrolizumab and 5 for nivolumab, but if neither was available, then the cutoff value or closest available data from the respective clinical trial was used. Descriptive calculations and specific statistical testing were conducted using SAS version 9.4 (SAS Institute Inc, Cary, NC), but all figures were created using Microsoft Excel and Microsoft PowerPoint.

## Results

**Figure 1.**
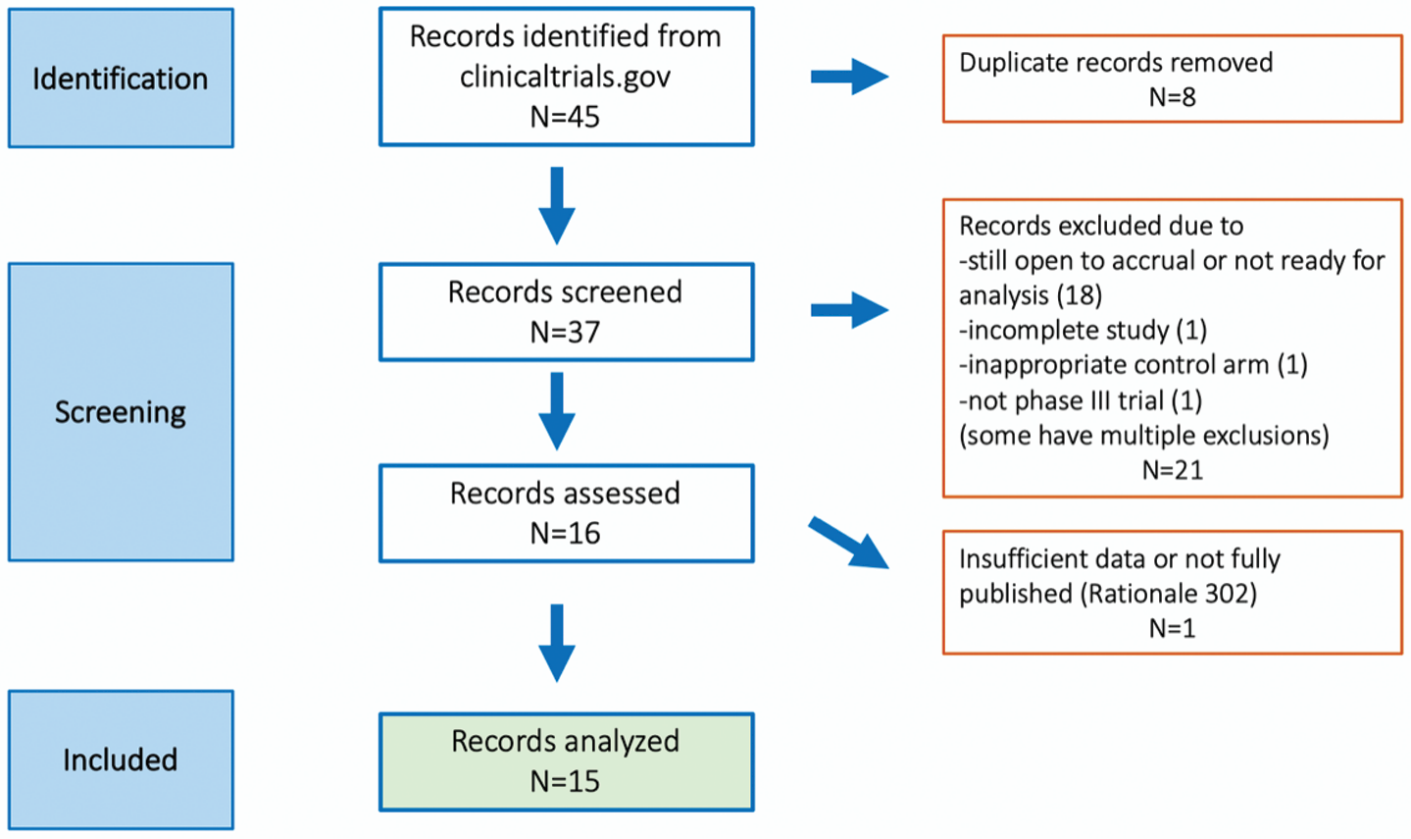
CONSORT diagram.

At the time of data cutoff, there were 45 records identified from clinicaltrials.gov, and 37 records were screened after removing duplicate records. Sixteen records fully reviewed and 15 unique trials were included in the final analysis (Fig. 1). Among the 15 studies, 7 are pembrolizumab, 6 are nivolumab, and 2 are avelumab trials (Table 1). Seven are first-line metastatic trials (including one maintenance trial), 7 are later-line metastatic trials, and one is an adjuvant trial. All studies used superiority over non-inferiority analysis. Six enrolled esophageal cancer, and 9 enrolled gastric cancer. All included GE junction cancers. The 15 clinical trials ranged from 94 to 1581 participants (Table 1). The reported median overall survival ranged from 4.6 to 17.5 months from these trials, and objective response rate ranged from 2.2% to 74%.

**Table 1.** Baseline characteristics (*N* = 15).

Characteristic	*n* (%)
Drug	
Pembrolizumab	7 (46.7%)
Nivolumab	6 (40%)
Avelumab	2 (13.3%)
Cancer type	
Esophageal	6 (40%)
Gastric	9 (60%)
Line of therapy	
First-line metastatic	7 (47%)
Second-line metastatic	5 (33.3%)
Third-line metastatic	2 (13.3%)
Adjuvant	1 (6.6%)
Blinding design	
Double blinded	6 (40%)
Open label	9 (60%)
Randomization scheme	
1:1	11 (73.4%)
1:1:1	2 (13.3%)
2:1	2 (13.3%)
Median (range)	
Sample size	628 (94-1581)
Median overall survival (months)	10.4 (4.6-17.5)
Gain in overall survival (months)	1.4 (-0.5-3.5)
Median progression-free survival (months)	2.9 (1.4-22.4)
Objective response rate (%)	20.3 (2.2-74.4)

**Figure 2.**
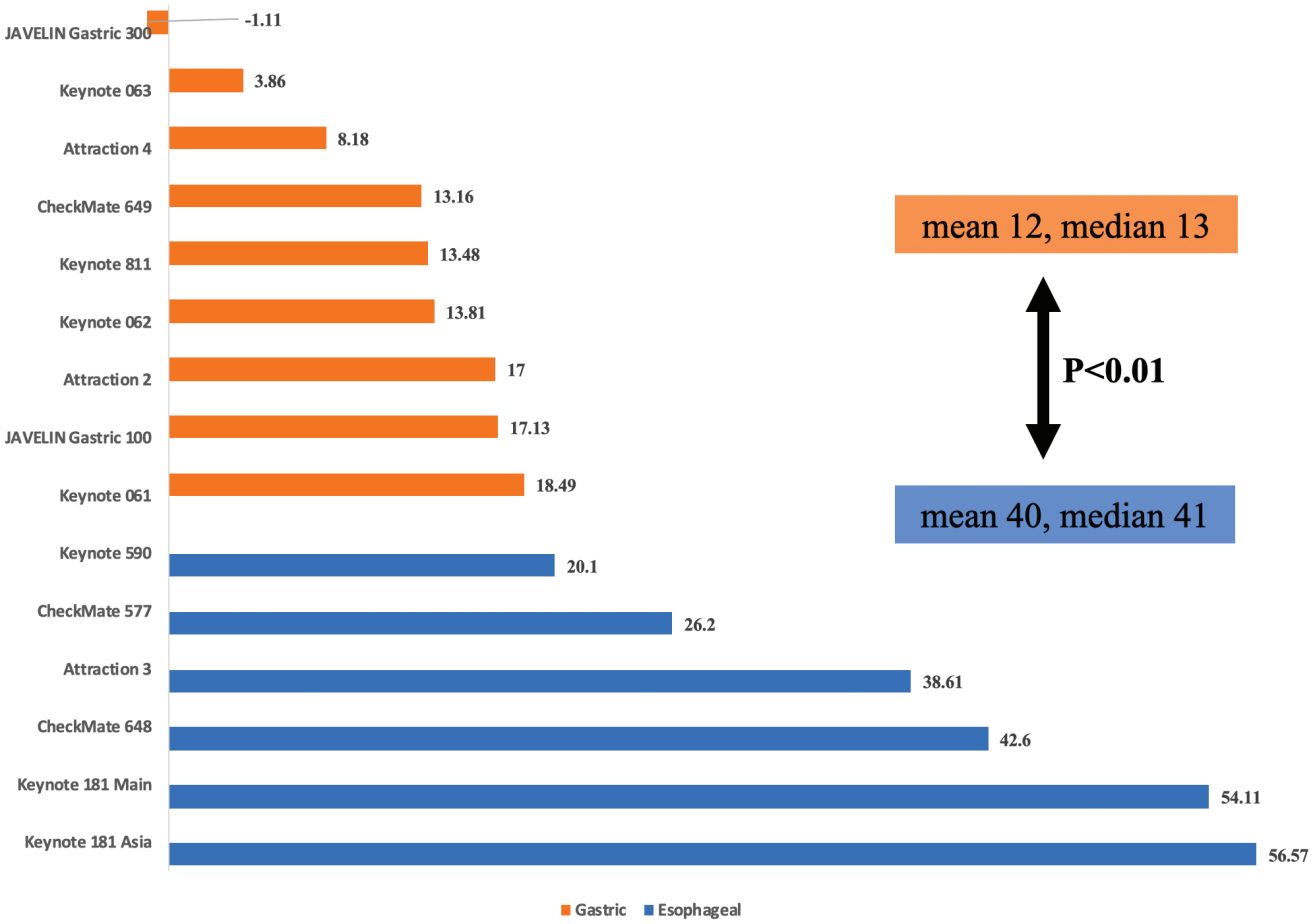
ASCO NHB Scores for esophageal vs. gastric cancer trials.

When comparing esophageal and gastric cancer trials, the ASCO Net Health scores was higher among esophageal cancer trials (mean 40, range 20.1-56.6) compared to gastric cancer trials (mean 12, range −1.1-18.4), *P* < .01 (Table 2; Fig. 2). In fact, all 6 esophageal cancer trials had scores exceeding 20, whereas all 9 gastric cancer trials were under 20 (Fig. 2). Likewise, for ESMO MCBS, all 6 esophageal cancer trials scored higher than 1, and all but 2 gastric trials scored 1. (*P* < .05, Table 2). When analyzing individual outcome endpoints, the 2 groups had similar magnitude of efficacy outcomes like progression-free survival, and response rates. The gain in overall survival was different, with mean 2.7 months (range 2.5 to 3.5) for esophageal and mean 0.62 (range −0.50 to 2.2), *P* = .014. The distribution of line of therapy and specific PD-L1 drug are also similar. When including data from pembrolizumab monotherapy (KEYNOTE 062) and nivolumab plus ipilimumab (CHECKMATE 648) for first-line metastatic setting, the ASCO Net Health scores and ESMO MCBS remained higher among esophageal than gastric cancer trials (Supplement). Details of all 15 studies are summarized in Table 3.

**Table 2.** ASCO Net Health Scores and ESMO Scale Comparison by Cancer (*N* = 15)

	Esophageal (*n* = 6)	Gastric (*n* = 9)	*P*-value
ASCO Net Health Scores, mean (range)	39.7 (20.1-56.6)	11.6 (−1.1 to 18.5)	<.01
Median overall survival, months, mean (range)	11.1 (9.3-13.2)^1^	10.1 (4.6-17.5)^2^	.62
Gain in overall survival, months, mean (range)	2.7 (2.5-3.5)^1^	0.6 (−0.5 to 2.2)^2^	.014
Median progression-free survival, months, mean (range)	6.9 (1.7-22.4)	4.4 (1.4-10.9)^2^	.57
Objective response rate, %, mean (range)	29.9 (17.1-47)^1^	32.6 (2.2-74.4)	.79
ESMO scale, *n* (%)			
1	0	7 (77.8%)	.011
2	5 (83.3%)	2 (22.2%)	
3	0	0	
4	1(6.7%)	0	

^1^No response rate or overall survival data from CHECKMATE 577.

^2^No survival data from KEYNOTE 811.

**Table 3.** Key details of included anti-PD1 and anti-PD-L1 immune checkpoint inhibitor phase III trials.

Study name	Registration # (PubMed PMID)	Cancer type	Experimental treatment	Control arm	Key reported outcomes
Pembrolizumab					
KEYNOTE 590	NCT03189719 (34454674)	Esophageal (all histology), 1st-line advanced/metastatic	5-Fluorouracil (5FU) and cisplatin with pembrolizumab	5FU and cisplatin with placebo	Median OS 13.5 vs. 9.4 months, and median PFS 7.5 vs. 5.5 months in PD-L1 CPS ≥10; ORR 45% in all patients
KEYNOTE 181 Asia	NCT03933449 (34973513)	Esophageal (all histology), 2nd-line advanced/metastatic	Pembrolizumab	Docetaxel, paclitaxel, or irinotecan	Median OS 10.0 months vs. 6.5 months, median PFS 2.3 vs. 3.1 months, and ORR 17%, in squamous histology
KEYNOTE 181	NCT02564263 (33026938)	Esophageal (all histology), 2nd-line advanced/metastatic	Pembrolizumab	Docetaxel, paclitaxel, or irinotecan	Median OS 9.3 vs. 6.7 months, median PFS 2.6 vs. 3.0 months, and RR 21.5% in PD-L1 CPS ≥10
KEYNOTE 061	NCT02370498 (29880231)	Gastric/GE junction adenocarcinoma, 2nd-line advanced/metastatic	Pembrolizumab	Paclitaxel	Median OS 9.1 vs. 8.3 months and median PFS 1.5 vs. 4.1 months in all patients; ORR 16% in PD-L1 CPS ≥1
KEYNOTE 063	NCT03019588 (34878659)	Gastric/GE junction adenocarcinoma (PD-L1 CPS ≥1), 2nd-line advanced/metastatic	Pembrolizumab	Paclitaxel	Median OS 8 vs. 8 months, median PFS 2 vs. 4 months, and ORR 13%
KEYNOTE 811	NCT03615326 (34912120)	Gastric/GE junction adenocarcinoma (HER2+), 1st-line unresectable/metastatic	Chemotherapy with trastuzumab and pembrolizumab	5FU and cisplatin, or capecitabine and oxaliplatin (CAPOX), with trastuzumab	ORR 74% vs. 52% and disease control 96% vs. 89%
KEYNOTE 062	NCT02494583 (32880601)	Gastric/GE junction adenocarcinoma (HER2−, PD-L1 CPS ≥1), 1st-line advanced/metastatic	Chemotherapy with pembrolizumab (also has pembrolizumab monotherapy arm)	5FU or capecitabine, and cisplatin, with placebo	Median OS 12.5 vs. 11.1 months, median PFS 6.9 vs. 6.4 months, and ORR 49%
Nivolumab					
CHECKMATE 648	NCT03143153 (35108470)	Esophageal, (squamous), 1st-line unresectable, recurrent, or metastatic	5FU and cisplatin with nivolumab (also has nivolumab with ipilimumab arm)	5FU and cisplatin	Median OS 15.4 vs. 9.1 months, median PFS 6.9 vs. 4.4 months, and ORR 53% in PD-L1 CPS ≥1
CHECKMATE 649	NCT02872116 (34102137)	Esophageal, GE junction, gastric (HER2− adenocarcinoma), 1st-line unresectable/metastatic	5FU or capecitabine, and oxaliplatin (FOLFOX or CAPOX), with nivolumab	FOLFOX or CAPOX	Median OS 14.4 vs. 11.1 months, median PFS 7.7 vs. 6.0 months, and ORR 60% in CPS ≥5
ATTRACTION 4	NCT02746796 (35030335)	GE junction, gastric (HER2− adenocarcinoma), 1st-line recurrent/advanced	Capecitabine or S-1 and oxaliplatin (CAPOX or SOX) with nivolumab	CAPOX or SOX	Median OS 17.5 vs. 17.2 months, median PFS 10.5 vs. 8.3 months, and ORR 57%
ATTRACTION 3	NCT02569242 (31582355)	Esophageal (squamous), 2nd-line advanced/metastatic	Nivolumab	Docetaxel or paclitaxel	Median OS 10.9 vs. 8.4 months, median PFS 1.7 vs. 3.4 months, and ORR 19%
ATTRACTION 2	NCT02267343 (28993052)	Gastric/GE junction adenocarcinoma, 3rd-line recurrent/metastatic	Nivolumab	Placebo	Median OS 5.3 vs. 4.1 months, median PFS 1.6 vs. 1.5 months, and ORR 11%
CHECKMATE 577	NCT02743494 (33789008)	Esophageal (all histology), adjuvant after tri-modality therapy for resectable cancer	Nivolumab	Placebo	Median DFS 22.4 vs. 11.0 months
Avelumab					
Javelin Gastric 100	NCT02625610 (33197226)	Gastric/GE junction adenocarcinoma, maintenance after induction 1st-line chemotherapy	Avelumab	Continuation of FOLFOX or CAPOX	Median OS 10.4 vs. 10.9 months, and median PFS 3.2 vs. 4.4 months
Javelin Gastric 300	NCT02625623 (30052729)	Gastric/GE junction adenocarcinoma, 2nd-line unresectable, recurrent or metastatic	Avelumab	Irinotecan or paclitaxel	Median OS 4.6 vs. 5.0 months, median PFS 1.4 vs. 2.7 months, and ORR 2.2%

**Figure 3.**
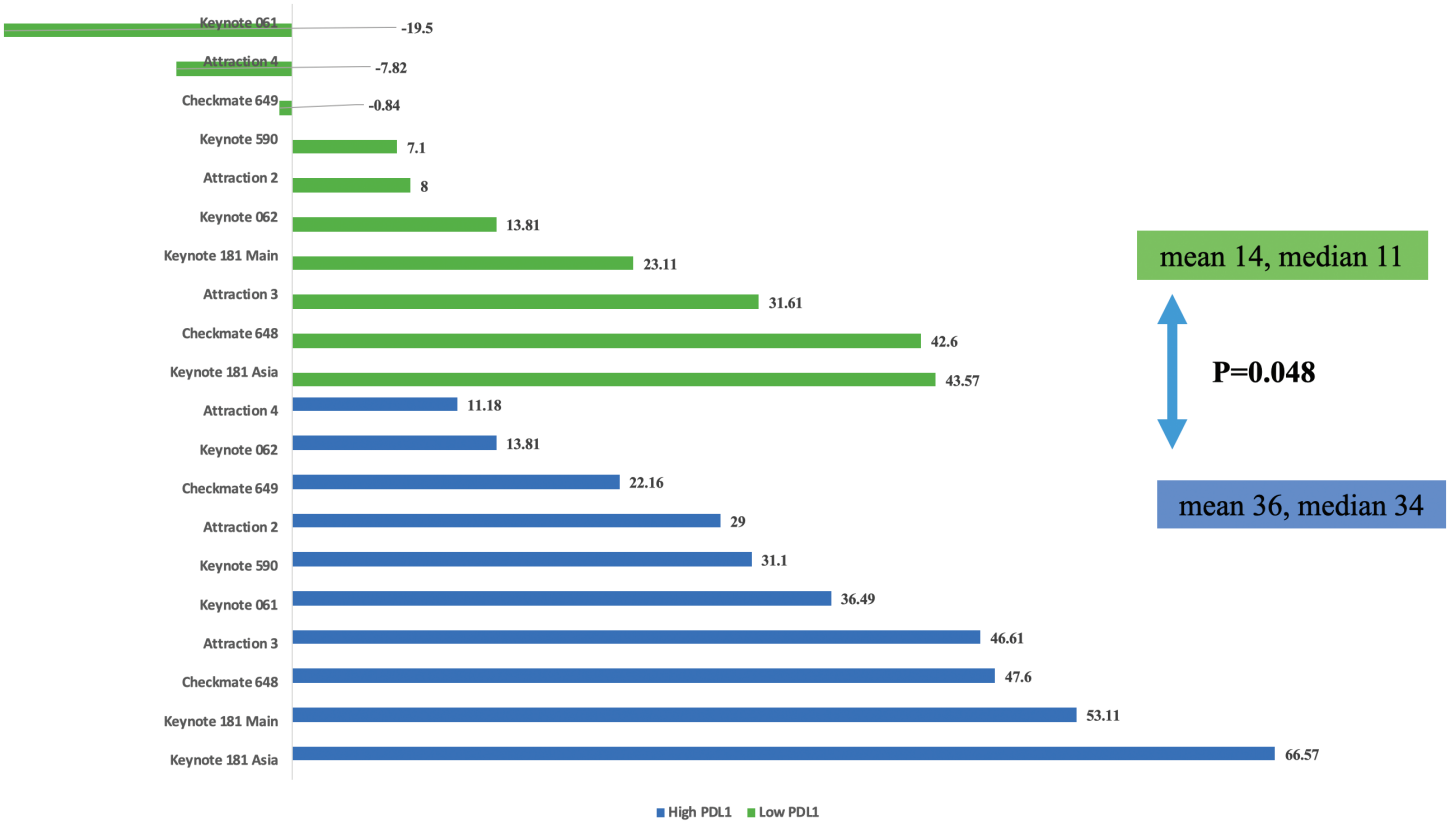
ASCO NHB Scores for high vs. low PD-L1.

With regards to PD-L1 expression, subgroups from trials with low PD-L1 scores in respective trials exhibited lower net health scores (mean 14.2, range −19.5 to -43.6) than subgroups from trials with high PD-L1 scores (mean 35.8, range 11.2 to 66.6, *P* = .048, Fig. 3). These differences were preserved when including data from pembrolizumab monotherapy (KEYNOTE 062) and nivolumab plus ipilimumab (CHECKMATE 648) for first-line metastatic setting (Supplement). About squamous histology, KEYNOTE 590 trial showed net health score of 21.1 and KEYNOTE 181 trial showed score of 46.1. Concerning adenocarcinoma histology, the same trials showed net health score 19.1 and 11.1, respectively, which are both numerically lower than squamous histology. Other esophageal cancer trials did not have sufficient data regarding histology subgroups for analysis.

Upcoming oncology trials not yet fully published or still accruing are presented in Supplement, which were all identified during the literature search and could be analyzed in the future.

## Discussion and Conclusion

Even though immunotherapy checkpoint inhibitors are FDA-approved for first-line metastatic esophageal and gastric cancer regardless of histology and PD-L1 expression, not all clinical scenarios will benefit from non-selective application of chemo-immunotherapy. In this umbrella review of most published drug registration trials of anti-PD1 and PD-L1 therapies for upper GI cancers, we showed the ASCO Net Health scores were consistently higher among esophageal cancer trials than gastric cancer trials. Differences were also seen in subgroups of high vs. low PD-L1 expression, and descriptively noted in squamous vs. adenocarcinoma histology. For example, KEYNOTE 181 trial, the hazard ratio for overall survival in for adenocarcinoma was 1.12 and CPS<10 was 1.00, both of which led to marginal net health scores in our analysis.^14^ Across all the trials, many adenocarcinoma, low PD-L1, and gastric cancer cohorts had net health scores lower than 30, though a target of 40 or lower had been previously suggested as potential threshold for marginal clinical value.^15^ All of these findings are relevant to shared decision-making regarding the use of immunotherapy checkpoint inhibitors in patients with gastric adenocarcinoma and/or with very low PD-L1 expression. If there is relevant financial burden or uncomfortable side effects, then there should be low threshold to hold off or discontinue the immunotherapy drug.

The phase II trial, KEYNOTE-059 trial (NCT02335411), first reported promising response rate of 11.6% in treatment-refractory gastric adenocarcinoma, which led to initial accelerated approval in the third-line setting for PD-L1 positive gastric adenocarcinoma.^8^ However, subsequent mandated phase III trials in 2 metastatic settings failed to show immunotherapy was better than gold-standard chemotherapy.^16,17^ These disappointing results led to withdrawal of single-agent pembrolizumab approval in the third-line setting in 2021. It is possible that the marginal clinical value of immunotherapy in specific relevant patient cohorts were confirmed in these phase III trials that enrolled broad populations without enriching patients who are most likely to benefit.

Avelumab had early disappointing results that did not lead positive trials that could be granted FDA approval, and nivolumab initially had only accelerated approval for esophageal squamous cell carcinoma after prior chemotherapy.^18,19^ However, once histology and PD-L1 expression were shown to be predictive for response, their use in statistical analysis led to successful some chemo-immunotherapy trial results, such as CHECKMATE 649, which tested FOLFOX and nivolumab, and KEYNOTE 590, which tested 5FU/cisplatin and pembrolizumab. In fact, a systematic review showed PD-L1 and tumor mutation burden have high correlation with treatment response.^20^ Future trials should use these biomarkers when planning clinical trials to exclude patients who may not benefit from immunotherapy. Many of these trials continue to enroll patients with very low PD-L1 CPS, including score of 0, who are not expected to have clinical benefit evidenced from prior studies.^21,22^ However, some caution must be noted, as recent studies have also reported subjective interpretation among pathologists and technical differences among various commercial PD-L1 immunohistochemistry stains, especially with regards to CPS over TPS, could lead to poor inter-assay concordance.^23^

While many clinicians are excited about the promise of immunotherapy, for many cancers the expected response rate is still only modest across solid tumors.^24^ It is concerning that more people are commercially “eligible” for these therapies than people who are actually expected to benefit. The story of immunotherapy today, like the overall pattern of cancer therapy development, is one where there seems to be an increase in costs without proportional increase in benefit over time.^25^ If we insist on applying immunotherapy to broad populations because of it being “well tolerated”, then we should consider lower costs to payers and patients when using these drugs. Researchers have asked whether therapies with low clinical benefit scores should be cheaper,^26^ but in fact, there is no difference between benefit and price.^27^ All in all, clinicians should carefully describe potential clinical value of immunotherapy per cancer type, histology, and PD-L1 expression as well as other individual factors, and importantly they should advocate for appropriate pricing, institutional treatment guideline, and research trial design within their capacity.

Several recent studies are of interest to medical oncologists. The FDA has already approved the use of pembrolizumab for HER2+ esophageal and gastric adenocarcinoma due to impressive preliminary objective response rate results based on KEYNOTE 811 trial.^28,29^ Further follow-up regarding progression-free survival and overall survival will be important, and specific subgroup analysis will be important to understand for what cohorts the therapy might be of high versus marginal clinical value. CHECKMATE 648, similarly, had demonstrated promising overall survival benefit when comparing nivolumab and ipilimumab compared to chemotherapy alone for patients with squamous esophageal cancer regardless of PD-L1 expression, which led to FDA approval in May 2022.^30,31^ This improvement in survival was not associated with worsening adverse events.

While there is little financial incentive to reconsider conventional cytotoxic drugs, it is possible that triplet cytotoxic chemotherapy DOF (or FLOT) and FOLFIRINOX may be helpful in patients with metastatic esophageal or gastric cancer with low PD-L1 who are unlikely to respond to immunotherapy checkpoint inhibitors.^32,33^ Additional ASCO and ESMO value assessments can be applied to all of these studies to further validate results considered here.

There are several study limitations. First, GE junction cancers are sometimes included in both esophageal and gastric cancers, so it is difficult to separate them in this analysis. Second, the primary endpoint used for ASCO and ESMO assessments may not always be representative to the entire randomized population in the trial. This actually highlights the level of manipulation in the study design of many of the trials included here, as hierarchical testing prevents the pharmaceutical company from being penalized statistically. Third, ASCO and ESMO scores ultimately may vary between assessors, though at least 2 reviewers were used in the assessment of these results. While some of these studies did not achieve primary endpoint, all of these were informative in showing the range of clinical value of immunotherapy. Finally, the current analysis includes heterogenous populations with adjuvant and palliative settings, which could influence the magnitude of benefit, but both ASCO and ESMO assessments have been previously validated across treatment settings and cancer types.

In conclusion, this is the first study that used ASCO and ESMO value assessment tools across immunotherapy checkpoint inhibitor trials in esophageal and gastric cancer. We noted differences between cancer types and raised awareness of specific subgroups that may not benefit from immunotherapy checkpoint inhibitors despite current FDA approval. Our findings here hopefully would inform clinical guidelines and individual physician-patient discussions so that immunotherapy is not overused in cases where there is excessive side effects or financial toxicities. We also hope that future investigators will practice equipoise and focus further drug registrations trials in patients who are expected to benefit rather than the entire population.

## Supplementary Material

oyac187_suppl_Supplementary_Material

## Data Availability

The data underlying is article is publicly available
