# Supplementary material for: Application of Value Framework to Phase III Trials of Immune Checkpoint Inhibitors in Esophageal and Gastric Cancer
Source: Oncologist. 2023 Jan 18;28(1):40–7. doi: 10.1093/oncolo/oyac187 (PMC9847562; doi:10.1093/oncolo/oyac187)
Supplement: oyac187_suppl_Supplementary_Material [file oyac187_suppl_supplementary_material.docx]

**Supplement**

| **Supplement 1. Relevant baseline factors between esophageal and gastric cancer trials** | | | |
| --- | --- | --- | --- |
| N=15 | **Esophageal (N=6)** | **Gastric (N=9)** | **P value** |

| **Drug** |  |  |  |
| --- | --- | --- | --- |
| Pembrolizumab | 3 (50%) | 4 (44.4%) | .62 |
| Nivolumab | 3 (50%) | 3 (33.3%) |  |
| Avelumab | 0 | 2 (22.2%) |  |
| **Line of Therapy** |  |  |  |
| First-line metastatic | 2 (33.3%) | 5 (55.6%) | .34 |
| Second-line metastatic | 3 (50%) | 2 (22.2%) |  |
| Third-line metastatic | 0 | 2 (22.2%) |  |
| Adjuvant | 1 (16.7%) | 0 |  |

| **Supplement 2. ASCO Net Health Scores and ESMO Scale with inclusion of pembrolizumab monotherapy (KEYNOTE 062) and nivolumab+ipilimumab arms (CHECKMATE 648)** | | | |
| --- | --- | --- | --- |
| N=17 | **Esophageal (N=7)** | **Gastric (N=10)** | **P value** |
|  | Mean (range) | Mean (range) |  |
| **ASCO Net Health Scores** | 40.4 (20.1-56.6) | 12.5 (-1.1-20.7) | <.01 |
| **Median overall survival** (months) | 11.4 (9.3-13.2)^1^ | 10.2 (4.6-17.5)^2^ | .53 |
| **Gain in overall survival** (months) | 2.6 (2.0-3.5)^1^ | 0.5 (-0.5-2.2)^2^ | <.01 |
| **Median progression-free survival** (months) | 6.3 (1.7-22.4) | 4.1 (1.4-10.9)^2^ | .47 |
| **Objective response rate** (%) | 29.6 (17.1-47)^1^ | 30.9 (2.2-74.4) | .56 |
| **ESMO scale** | N (%) | N (%) |  |
| 1 | 1 (14.3%) | 8 (80%) | .02 |
| 2  3  4 | 5 (71.4%)  0  1(14.3%) | 2 (20%)  0  0 |  |
| ^1^No response rate or overall survival data from CHECKMATE 577  ^2^No survival data from KEYNOTE 811 | | | |

| **Supplement 3. Selected list of incomplete, ongoing, or upcoming immune checkpoint inhibitor phase 3 trials** | | | |
| --- | --- | --- | --- |
| **Study Name** | **Registration #** | **Cancer Type** | **Experimental treatment** |
| Keynote 975 | NCT04210115 | Esophageal | Definitive chemo-radiation with fluorouracil, platinum, and pembrolizumab for non-metastatic, unresectable cancer |
| Leap 015 | NCT04662710 | Esophageal | FOLFOX/CAPOX, lenvatinib and pembrolizumab for first-line unresectable or metastatic cancer |
| Keynote 585 | NCT03221426 | Gastric | Perioperative FLOT or 5FU/cisplatin with pembrolizumab for resectable cancer |
| Keynote 859 | NCT03675737 | Gastric | 5FU and cisplatin/CAPOX with pembrolizumab for first-line unresectable or metastatic cancer |
| Ecog-Acrin 2174 | NCT03604991 | Esophageal | Neoadjuvant chemo-radiation with carboplatin, paclitaxel, and nivolumab |
| Attraction 5 | NCT03006705 | Gastric | Adjuvant CAPOX/S-1 and oxaliplatin and nivolumab |
| Skyscraper 07 | NCT04543617 | Esophageal | Consolidative atezolizumab +/- tiragolumab after definitive chemo-radiation |
| Skyscraper 08 | NCT04540211 | Esophageal | Cisplatin and paclitaxel with atezolizumab + tiragolumab for first-line unresectable or metastatic cancer |
| Kunlun | NCT04550260 | Esophageal, (squamous) | Chemo-radiation with durvalumab |
| Matternhorn | NCT04592913 | Gastric | Perioperative FLOT with durvalumab |
| Rationale 305 | NCT03777657 | Gastric | Platinum/fluoropyrmidine with tislelizumab |
| Rationale 306 | NCT03783442 | Esophageal (squamous) | Any chemo doublet with tislelizumab |
| Rationale 302 | NCT03430843 | Esophageal (squamous) | Second-line tislelizumab for metastatic cancer |
| Rationale 311 | NCT03957590 | Esophageal (squamous) | Tislelizumab with chemo-radiation |

**Supplement 4: ASCO NHB Scores for esophageal and gastric cancer trials with additional experimental arms from CHECKMATE 648 and KEYNOTE 062**


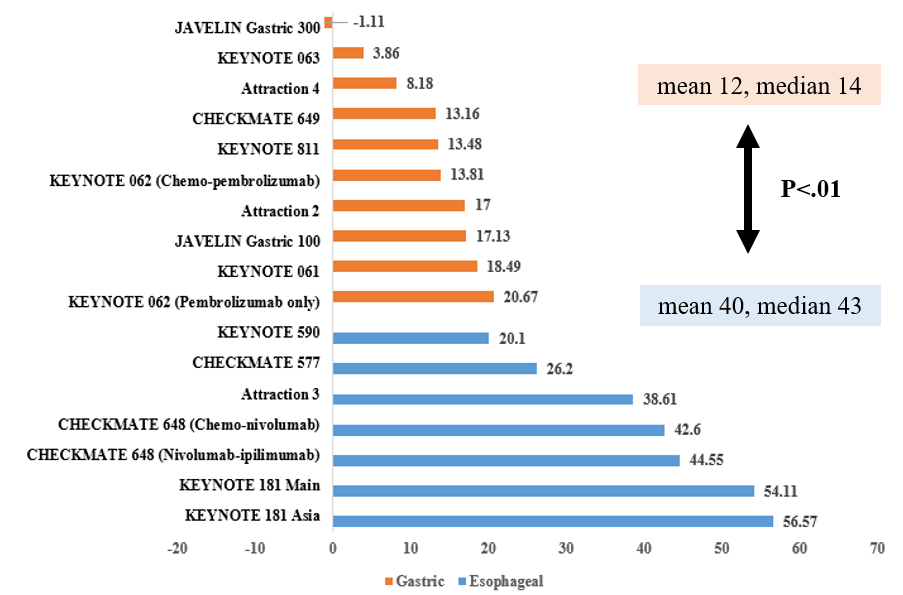


**Supplement 5: ASCO NHB Scores for high vs. low PD-L1 with additional experimental arms from CHECKMATE 648 and KEYNOTE 062**


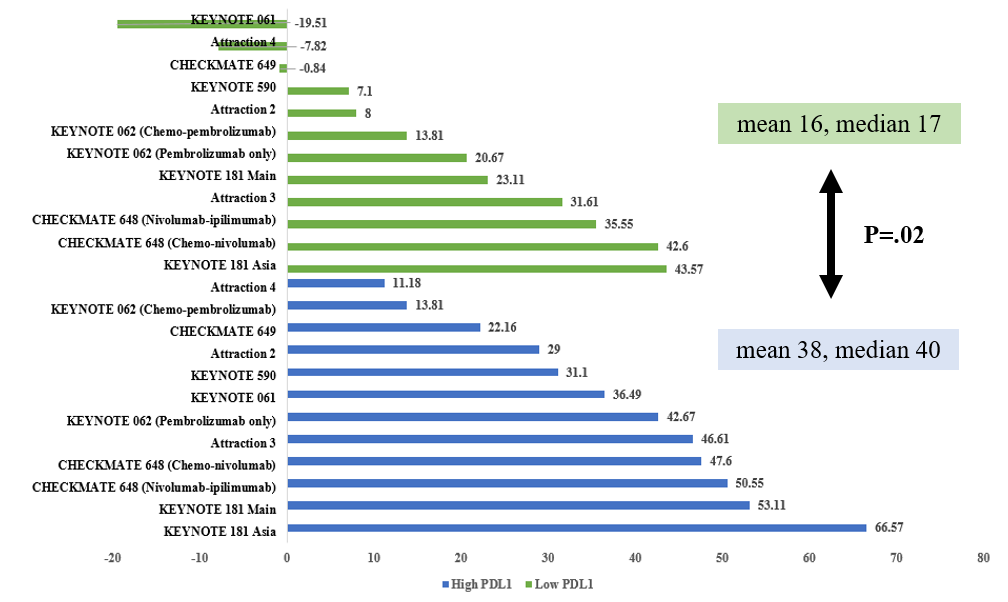


**Supplement 6: Reference list of 15 studies used for data extraction**

**^1-15^**

1. Bang YJ, Ruiz EY, Van Cutsem E, et al. Phase III, randomised trial of avelumab versus physician's choice of chemotherapy as third-line treatment of patients with advanced gastric or gastro-oesophageal junction cancer: primary analysis of JAVELIN Gastric 300. *Ann Oncol.* 2018;29(10):2052-2060.

2. Cao Y, Qin S, Luo S, et al. Pembrolizumab versus chemotherapy for patients with esophageal squamous cell carcinoma enrolled in the randomized KEYNOTE-181 trial in Asia. *ESMO Open.* 2022;7(1):100341.

3. Chung HC, Kang YK, Chen Z, et al. Pembrolizumab versus paclitaxel for previously treated advanced gastric or gastroesophageal junction cancer (KEYNOTE-063): A randomized, open-label, phase 3 trial in Asian patients. *Cancer.* 2022;128(5):995-1003.

4. Doki Y, Ajani JA, Kato K, et al. Nivolumab Combination Therapy in Advanced Esophageal Squamous-Cell Carcinoma. *N Engl J Med.* 2022;386(5):449-462.

5. Janjigian YY, Kawazoe A, Yañez P, et al. The KEYNOTE-811 trial of dual PD-1 and HER2 blockade in HER2-positive gastric cancer. *Nature.* 2021;600(7890):727-730.

6. Janjigian YY, Shitara K, Moehler M, et al. First-line nivolumab plus chemotherapy versus chemotherapy alone for advanced gastric, gastro-oesophageal junction, and oesophageal adenocarcinoma (CheckMate 649): a randomised, open-label, phase 3 trial. *Lancet.* 2021;398(10294):27-40.

7. Kang YK, Boku N, Satoh T, et al. Nivolumab in patients with advanced gastric or gastro-oesophageal junction cancer refractory to, or intolerant of, at least two previous chemotherapy regimens (ONO-4538-12, ATTRACTION-2): a randomised, double-blind, placebo-controlled, phase 3 trial. *Lancet.* 2017;390(10111):2461-2471.

8. Kang YK, Chen LT, Ryu MH, et al. Nivolumab plus chemotherapy versus placebo plus chemotherapy in patients with HER2-negative, untreated, unresectable advanced or recurrent gastric or gastro-oesophageal junction cancer (ATTRACTION-4): a randomised, multicentre, double-blind, placebo-controlled, phase 3 trial. *Lancet Oncol.* 2022;23(2):234-247.

9. Kato K, Cho BC, Takahashi M, et al. Nivolumab versus chemotherapy in patients with advanced oesophageal squamous cell carcinoma refractory or intolerant to previous chemotherapy (ATTRACTION-3): a multicentre, randomised, open-label, phase 3 trial. *Lancet Oncol.* 2019;20(11):1506-1517.

10. Kelly RJ, Ajani JA, Kuzdzal J, et al. Adjuvant Nivolumab in Resected Esophageal or Gastroesophageal Junction Cancer. *N Engl J Med.* 2021;384(13):1191-1203.

11. Kojima T, Shah MA, Muro K, et al. Randomized Phase III KEYNOTE-181 Study of Pembrolizumab Versus Chemotherapy in Advanced Esophageal Cancer. *J Clin Oncol.* 2020;38(35):4138-4148.

12. Moehler M, Dvorkin M, Boku N, et al. Phase III Trial of Avelumab Maintenance After First-Line Induction Chemotherapy Versus Continuation of Chemotherapy in Patients With Gastric Cancers: Results From JAVELIN Gastric 100. *J Clin Oncol.* 2021;39(9):966-977.

13. Shitara K, Özgüroğlu M, Bang YJ, et al. Pembrolizumab versus paclitaxel for previously treated, advanced gastric or gastro-oesophageal junction cancer (KEYNOTE-061): a randomised, open-label, controlled, phase 3 trial. *Lancet.* 2018;392(10142):123-133.

14. Shitara K, Van Cutsem E, Bang YJ, et al. Efficacy and Safety of Pembrolizumab or Pembrolizumab Plus Chemotherapy vs Chemotherapy Alone for Patients With First-line, Advanced Gastric Cancer: The KEYNOTE-062 Phase 3 Randomized Clinical Trial. *JAMA Oncol.* 2020;6(10):1571-1580.

15. Sun JM, Shen L, Shah MA, et al. Pembrolizumab plus chemotherapy versus chemotherapy alone for first-line treatment of advanced oesophageal cancer (KEYNOTE-590): a randomised, placebo-controlled, phase 3 study. *Lancet.* 2021;398(10302):759-771.
